# The Chemistry behind Chocolate Production

**DOI:** 10.3390/molecules24173163

**Published:** 2019-08-30

**Authors:** Veronika Barišić, Mirela Kopjar, Antun Jozinović, Ivana Flanjak, Đurđica Ačkar, Borislav Miličević, Drago Šubarić, Stela Jokić, Jurislav Babić

**Affiliations:** Josip Juraj Strossmayer University of Osijek, Faculty of Food Technology Osijek, Franje Kuhača 20, 31000 Osijek, Croatia

**Keywords:** chocolate, cocoa beans, Maillard reactions, polyphenols, pyrazines

## Abstract

Chocolate production is a complex process during which numerous chemical reactions occur. The most important processes, involving most of the reactions important for development of the proper chocolate flavor, are fermentation, drying and roasting of cocoa bean, and chocolate conching. During fermentation, formation of important precursors occurs, which are essential for further chemical reactions in the following processes of chocolate production. Roasting is one of the most important processes due to the occurrence of Maillard’s reactions, during which aroma compounds are formed. In this paper, we have reviewed the most important chemical reactions that occur with proteins, carbohydrates, lipids, and polyphenols. Additionally, we present other components that may be naturally present or form during the production process, such as methylxanthines, aldehydes, esters, ketones, pyrazines, acids, and alcohols.

## 1. Introduction

Chocolate is a product widely consumed by all generations. It is rich in fat, proteins, carbohydrates, polyphenols and other bioactive compounds [1]. Cocoa beans are main ingredient for production of chocolate. The chocolate production process consists of fermentation, drying, roasting, grinding of cocoa beans, mixing of all ingredients (cocoa mass, sugar, cocoa butter, emulsifiers, aroma, and milk components if needed), conching, and tempering. Major chemical reactions occur during fermentation, drying, roasting of cocoa beans, and conching of chocolate mass. These reactions are the most important for flavor and aroma development [2].

Fermentation of cocoa beans is a process in which growth of yeasts and bacteria occur in the pulp and it is conducted at cocoa plantations as a part of the cocoa beans’ production. In this stage, breakdown of sugars and mucilage occurs. It consists of three phases:

(1) The first phase is dominated by anaerobic yeasts and lasts for 24–36 h. In this phase, low content of oxygen and low pH (<4) are present.
(2) The second phase is dominated by lactic acid bacteria. They are present from the beginning but become active between 48–96 h.
(3) Third phase is dominated by acetic acid bacteria when aeration increases. During this last phase, an exothermic reaction (conversion of alcohol into acetic acid) occurs, responsible for a temperature rise (50 °C or higher).

These phases of cocoa bean fermentation are described in detail in Beckett et al. [3]. Fermentation is the key stage for the production of precursors for development of proper chocolate aroma [3,4,5].

Drying of cocoa beans is conducted after fermentation to lower the moisture content and complete oxidative processes induced during fermentation of cocoa beans. After drying, cocoa beans have 6–8% moisture. Low moisture content prevents mold growth and makes the beans more stable for transport and storage [2,3]. 

Roasting of cocoa bean is usually conducted in a chocolate factory as a first stage of chocolate production. It is a high temperature process, usually conducted at temperatures between 120 and 140 °C, which is important for the occurrence of Maillard reactions. Roasting reduces contents of undesirable components, produces chocolate-specific aroma and flavor, and decontaminates the cocoa beans. In this phase, all precursors formed in the previous phases react and form numerous compounds [2,3,5].

Grinding and mixing of all chocolate ingredients are very important for achievement of the right particle size of all ingredients. The main ingredients used in chocolate production are cocoa liquor (obtained by grinding cocoa beans), cocoa butter (obtained by pressing cocoa liquor), sugar, and milk (in the case of milk chocolate) [3].

Conching is a mixing and heating treatment that is conducted to produce liquid chocolate (all solid particles are coated with fat), evaporate volatile acids, achieve a proper viscosity, remove excess moisture, and develop a desirable color [5,6,7].

Tempering is a process by which to obtain a stable product. Tempering is conducted thermally and results in stable and consistently sized crystals of cocoa butter which then affect growth of a stable crystalline network during cooling [3].

This paper reviews the major chemical reactions that occur during the chocolate production process and gives insights into the latest understandings of the most important components of cocoa beans and chocolate.

## 2. Proteins

### 2.1. Fermentation

**Figure 1:**
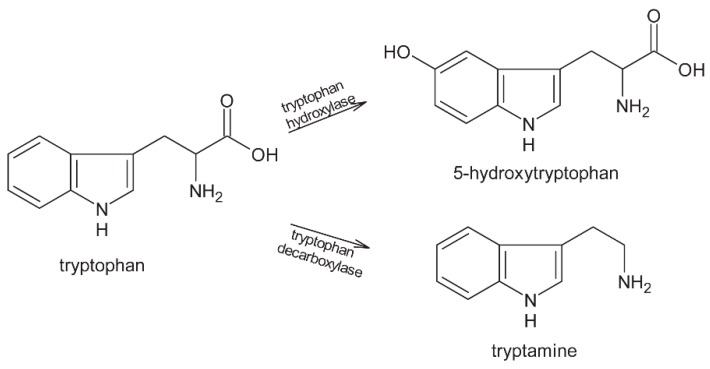
Possible transformation of tryptophan during fermentation of cocoa beans.

Proteins make 10–15% of the dry weight of unfermented cocoa beans. The most important are albumin (water soluble), prolamin (alcohol soluble), globulin (vicilin-type, salt soluble), and glutein (soluble in dilute acids and alkali) [8,9]. Fermentation is a process important for supplying precursors for further reactions. The low pH that develops during fermentation activates the endogenous enzymes carboxypeptidase and aspartic endoprotease. Carboxypeptidase and aspartic endoprotease are most active at pH 3.8 to 5.8 [8,10,11]. These enzymes hydrolyze proteins and, during fermentation, there is an increase in free amino acids and oligopeptides. The highest activity of these enzymes occurs shortly after bean death (24–48 h) [8,12,13]. Unfermented beans have a lower content of free amino acids then fermented beans. The ratio of acidic/hydrophobic/basic/other free amino acids in unfermented beans depends mostly on the origin and type of the beans. Regardless of the aforementioned ratio, during fermentation, the content of hydrophobic free amino acids increases and the content of acidic amino acids decreases. Hydrophobic amino acids, especially leucine, alanine, and phenylalanine, released during fermentation are responsible for development of Strecker aldehydes and pyrazines [8,13]. This was confirmed by the research of Adeyeye et al. [4] who discovered that fermented beans have a higher content of crude protein when compared with unfermented beans. They showed that phenylalanine and leucine are the predominant amino acids after fermentation [10]. Free tryptophan (which can be present as protein-bound as well), is transformed during fermentation into biogenic amines (5-hydroxytryptamine and tryptamine) [9]. Although the exact mechanism of tryptamine formation in cocoa beans has not been revealed, Oracz and Nebesny [14] proposed a mechanism of its enzymatic formation from tryptophan (Figure 1) and do Carmo Brito et al. [15] reported that the level of tryptamine decreases in the first four days of fermentation, only to slowly increase to virtually the same level by the sixth day. The presence of small amounts of tryptamine in raw cocoa beans has also been reported, as well as differences regarding variety (due to different composition of the bean) and origin (due to differences in cultivation conditions and microflora involved in the fermentation process) of cocoa beans [14]. Therefore, it is yet to be revealed whether the process of tryptamine synthesis from tryptophan is spontaneous, or whether endogenous enzymes or enzymes of microflora during fermentation are involved. Since biogenic amines are known to be the most abundant in fermented foods, it is most likely that fermentation of cocoa beans and microflora involved in the process are the most relevant in tryptamine synthesis.

Hydrophobic oligopeptides that are formed from hydrophobic amino acids mentioned above become substrates for serine carboxy-(exo) peptidase. Hydrophilic oligopeptides and hydrophobic free amino acids (chocolate aroma precursors) are obtained by the activity of carboxypeptidase from hydrophobic oligopeptides [16]. During fermentation, precursor compounds derived from free amino acids are released. 3-methylbutanol, phenylacetaldehyde, 2-methyl-3-(methyldithio)furan, 2-ethyl-3,5-dimethyl, and 2,3-diethyl-5-methylpyrazine are among the most important [5]. Also, decarboxylation of amino acids during fermentation results in formation of biogenic amines (2-phenylethylamine, 2-methylpropylamine, and 2- and 3-methylbutylamine). These components are already present in unfermented beans, but during fermentation, decarboxylases lead to an increase in the content of bioactive amines [8,10].

### 2.2. Roasting

**Figure 2:**
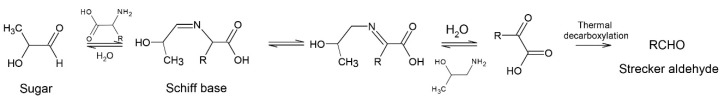
Strecker-type reaction for formation of aldehydes during roasting of cocoa beans.

Since roasting is conducted at high temperatures, precursors, which were obtained by protein degradation during fermentation, are subjected to chemical reactions. The most important is the Maillard reaction between the carbonyl group of reducing sugars and the amino group from amino acids or proteins. Through these reactions, cocoa obtains its chocolate specific aroma and flavor [4]. The condensation product that results from these reactions is transformed by Amadori rearrangement to 1-deoxy-2-ketosyl [2,4]. Roasting is more effective in the generation of amines and aldehydes than fermentation. Strecker-type reactions form aldehydes in roasted beans from amino acids (Figure 2) [10,17]. In addition, Oracz and Nebesny [18] reported that at higher roasting temperatures (135 and 150 °C), Maillard reactions advance to produce melanoidins, high-molecular weight (HMW) compounds that confer the specific brown color and texture to cocoa beans. They distinguish three types of melanoidins: “(1) polymers consisting of repeating furans or pyrrole units linked via polycondensation reactions; (2) carbohydrate skeletons built mainly from sugar degradation products polymerized through aldol-type condensation reactions and possibly linked by nitrogenous residues; and (3) protein-like structures arising from crosslinks between proteins and low-molecular weight (LMW) compounds formed in the advanced stages of Maillard reactions”. They also imply that, since the carbohydrate content in cocoa beans is relatively low (unlike coffee beans, for example), it is more likely that products of lipid oxidation, rather than sugars, are involved in reactions with proteins to form HMW melanoidins. However, this link is not well established by the results of their research and should be further investigated.

### 2.3. Other Processes

Casein is one of the most important proteins in milk. It can be present in milk chocolate and form an interface between solid and fatty components, meaning that casein can act as an emulsifier [2,3]. During conching, caramelization of lactose in milk chocolates and Maillard reactions with milk proteins occur [19]. Conching does not change the amino acid content of chocolate [20].

## 3. Carbohydrates

### 3.1. Fermentation

Unfermented cocoa beans contain 2–4% of low molecular weight carbohydrates and their derivatives, mostly sucrose (90% of total sugars), glucose, fructose, galactose, sorbose, arabinose, xylose, mannitol, and inositol. Polysaccharides (starch (4–6%), pectins, cellulose (2–3%) etc.) are also present in beans at a proportion of approximately 12%. Redgwell and Hansen [21] reported that cell wall polysaccharides (CWP) include cellulose (28% of CWP), pectic polysaccharides (containing galactose as the predominant side-chain sugar (9% of CWP) and containing arabinose as the predominant side-chain sugar (52% of CWP)), hemicellulosic polysaccharides (xyloglucan (8% of CWP), and gallactoglucomannan (3% of CWP)). They also reported that fermentation does not influence CWP. Sucrose is the main subject of reactions during fermentation. Sugars from pulp are converted to acids during fermentation. These acids move into the beans and lower their pH, which leads to breakdown of storage cells. There are two types of storage cells in the cotyledon: polyphenolic cells, which consist of a large vacuole that is filled with polyphenols and alkaloids; and lipid-protein cells, which have small lipid and protein vacuoles tightly packed inside the cytoplasm. Yeasts present during fermentation convert the present sugars to alcohol, which benefits development of lactic acid bacteria [7,8,12,22]. The alcohol is converted to acetic acid under aerobic conditions and the activity of acetic acid bacteria.

Sucrose is hydrolyzed into fructose and glucose (reducing sugars) due to invertase activity [8]. Megías-Pérez et al. [23] found seyllo-inositol (trace—504.9 μg/g in unfermented and 15.5–491.9 μg/g in fermented bean), 1-kestose (36.1–133.5 μg/g in unfermented and traces—115.5 μg/g in fermented bean), and 6-kestose (no reported values) for the first time. The content of these compounds was related to the origin of the cocoa beans. They also reported presence of galactinol in unfermented beans (traces—1970.4 μg/g). Since they were unable to detect galactinol in fermented beans, they related this compound to the fermentation status of the cocoa beans.

Reducing sugars obtained during fermentation are the main compounds of aroma development in the later processes of chocolate production.

### 3.2. Drying

During drying, reducing sugars obtained during fermentation react with other components of cocoa beans. These reactions are actually Maillard reactions between free amino acids and reducing sugars [16].

### 3.3. Roasting

**Figure 3:**
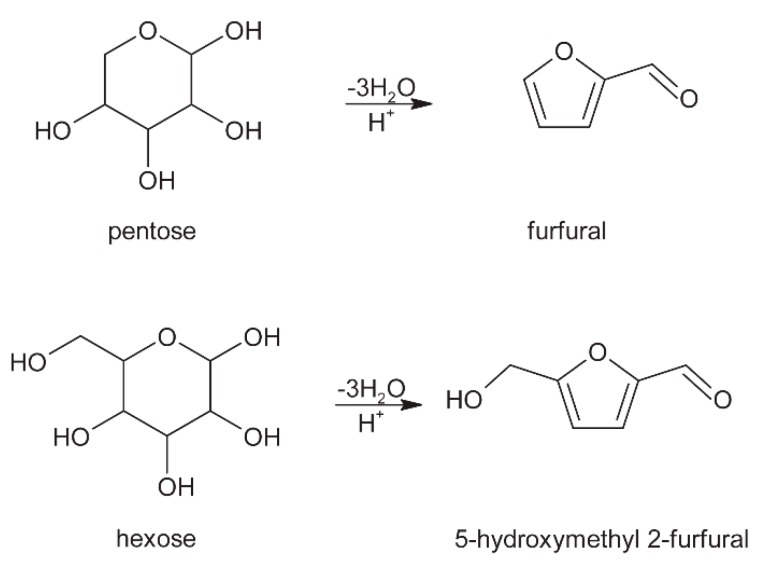
Dehydration of sugars.

Roasting of cocoa beans leads to caramelization of the present sugars. Caramelization occurs during heating of carbohydrates (especially sucrose and reducing sugars), where high temperature causes dehydration of sugars. During caramelization, molecular degradation of sugars leads to the development of aroma compounds. Also, during roasting, dehydration of sugars can occur which produces furfural and its derivatives (Figure 3) [2,17].

Finished chocolate contains about 50% sugar, which is mainly added sucrose, but it can also be lactose (in milk chocolate). In the last few years, fructose, sorbitol, polydextrose, etc. have been added in chocolates [2].

After chocolate production, sugar bloom on the chocolate surface may appear. This happens due to high humidity when moisture condenses on the surface of chocolate. Sugar from chocolate is extracted out on the surface and recrystallizes after the water is removed [12].

Noor-Soffalina et al. [24] found that cocoa beans with higher polyphenol content have lower sugar content. This phenomenon is most likely due to binding of carbohydrates to polyphenols. Hydrogen bonding of these components is created between hydroxyl groups and through hydrophilic interactions. Tannins are more likely to bond with carbohydrates because they are rich in closely set hydroxyl groups [25].

## 4. Lipids

Cocoa beans contain 50–58% fats, 97–98% of which are triacylglycerols (TAGs). TAGs consist of 24.1–27.1% palmitic acid, 32.9–37.6% stearic acid, and 32.7–37.6% oleic acid, and low amounts of linoleic acid (2.3–3.7%). Fatty acid composition depends on origin, variety of beans, growing season, and method of cultivation [8,26]. There are differences in cocoa butter considering origin and bean type. Softer cocoa butter has a higher content of 1-palmitoyl-2-3-dioleoyl-glycerol (POO) and 1-stearoyl-2-3-dioleoyl-glycerol (SOO), while harder cocoa butter has increased content of saturated fatty acids [26]. Sirbu et al. [26,27] found that only unfermented beans contain TAG containing hydroxyl-allyl fatty acid substituent by which they can be differentiated from fermented beans. They named this newly identified component cacaoic acid and stated that during fermentation, degradation or transformation of this component may occur. Future research is needed to further explore this new component and its fate during fermentation.

Fats which are rich in symmetrical monounsaturated triglycerides, such as cocoa butter, show a high degree of polymorphism [3]. Regardless of the TAG composition, cocoa butter can exist in six polymorphs: γ, α, β’(III), β’(IV), β(V), and β(VI) which have melting points of 17.3, 23.3, 25.5, 27.5, 33.8, and 36.3, respectively, and can transform from one to another, depending on temperature and time. The most desirable form in chocolate is β(V) which melts at 29–31.5 °C. This form is obtained by a properly conducted tempering process. Fat bloom is one of main problems in finished chocolate. In well-tempered chocolates, it can occur from transition of β(V) to more stable β(VI) form [3,28].

Milk fat is present in milk chocolates and consists of 98% TAGs, 2% phosphoglycerides, and traces of diacylglycerides and sterols [8]. In milk chocolates, conjugated linoleic acid (CLA) is also detected in amounts between 0.07 and 0.18%. The origin of CLA in milk chocolate is probably from milk, where it is recognized as group of positional and geometrical isomers of 18-carbon conjugated dienoic acid. CLA is known for anticarcinogenic activity [29,30,31]. Milk fat is known for its inhibition of fat bloom. It lowers solid fat content and slows down degree of cocoa butter crystallization. Milk fat is β’ stable and delays transition of β(V) to β(VI) form of cocoa butter because of its crystal size, better consistency of crystals, and smaller distance between unit cells [32].

## 5. Polyphenols

**Figure 4:**
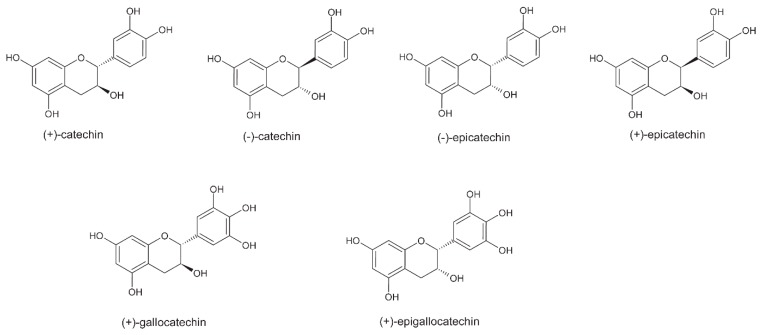
Structure of main flavan-3-ols present in cocoa and chocolate.

It is well known that cocoa and chocolate are rich in polyphenols. Unfermented cocoa beans contain 12–18% polyphenols of the dry weight of beans on average [33]. Cocoa polyphenols consist of approximately 37% flavan-3-ols, 4% anthocyanins, and 58% proanthocyanidins. These compounds are stored in polyphenolic cells in unfermented beans. In this form, they confer a white to deep purple color to unfermented beans. Catechins make up approximately 29–38% of total polyphenols. In cocoa and chocolate, they are represented by (−)-epicatechin, (+)-catechin, (+)-gallocatechin, and (−)-epigallocatechin (Figure 4). (−)-epicatechin makes up up to 35% of total polyphenols. They are essential for bean flavor and color development [5].

Anthocyanins in cocoa are represented by cyanidin-3-α-l-arabinoside and cyanidin-3-β-d-galactoside [7]. Procyanidins are represented by dimers, trimers, and oligomers of flavan-3,4-diols. Epicatechin is the main subunit, linked by 4–8 or 4–6 bonds [5,7]. Procyanidins can exist in A or B form [29,34]. All these compounds contribute to astringency and bitterness specific of unfermented cocoa [1].

### 5.1. Fermentation

**Figure 5:**
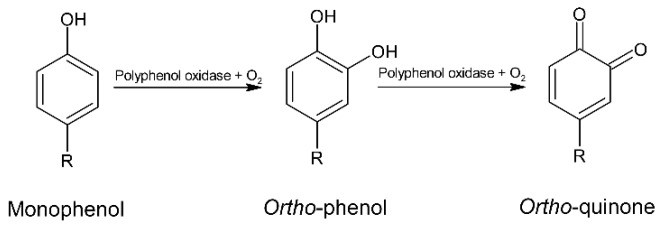
Oxidation of phenols by polyphenol oxidase.

Polyphenolic cells are destructed during fermentation and polyphenols exude from the cells. During fermentation, aerobic oxidation of polyphenols occurs. This reaction is carried out by polyphenol oxidase (Figure 5), which is released because of cotyledon changes affected by fermentation. The results of this process are reduced bitterness and astringency and increased brown color [5,35]. During oxidation, polyphenols react with proteins and are converted into insoluble forms [8].

Catechin content is mainly decreased during fermentation (more than 90% of initial content). It is found that (−)-epicatechin is significantly decreased [7]. During fermentation, procyanidin A2 epimerizes to procyanidin F2. F1 and F2 procyanidins are specific to fermented cocoa beans [35].

Hydrolysis of glycosides (mainly anthocyanins), nonenzymatic oligomerization of catechin, and transfer of proanthocyanidins into a more complex form lead to brightening of the cotyledon. Since the decrease of anthocyanin content during fermentation is significant, it is considered as a good fermentation index [7,36,37].

### 5.2. Drying

Drying also decreases the content of polyphenols in beans. This reduction is catalyzed by polyphenol oxidase, after which, new aroma compounds arise alongside the formation of brown color, and bitterness and astringency are reduced [7,16].

### 5.3. Roasting

**Figure 6:**
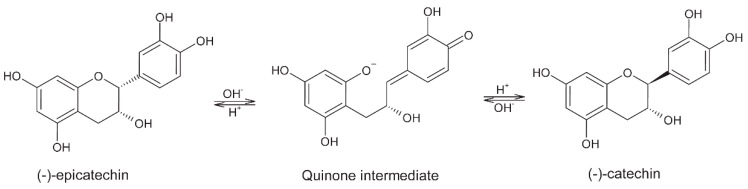
Epimerization of (−)-epicatechin to (−)-catechin.

As previously mentioned, roasting is conducted at high temperatures and thus significantly affects the content and composition of thermolabile molecules such as polyphenols. Reduction of total polyphenol content during roasting is observed in numerous studies [8,38,39]. Aprotosoaie et al. [7] observed that the decrease of high molecular weight proanthocyanidins is more pronounced. During roasting, the ratio of epicatechin/catechin is also changing. Ioannone et al. [40] concluded that the epicatechin/catechin ratio changed during roasting and that the content of (−)-epicatechin decreased. Stanley et al. [41] also observed a decrease of (−)-epicatechin but they found that it is subjected to epimerization and that the content of (−)-catechin was increased (Figure 6). Also, high temperatures can induce (+)-catechin epimerization to (+)-epicatechin [42]. The content of procyanidin B2 is also decreased during roasting, while procyanidin B1 is increased [39]. Proanthocyanidins are also subjected to epimerization, so it is noted that after roasting, the content of large proanthocyanidins is increased [38,41]. During roasting, a decrease of (−)-epicatechin and procyanidin B2 and an increase of (+)-catechin and procyanidin B1 was observed [39,41].

### 5.4. Conching

In the initial stage of conching, volatile polyphenols are lost due to evaporation, together with water and short-chain fatty acids. It has been established that the content of volatile polyphenols is reduced by 80% in this process [5]. During conching, oxidation of tannins occurs as well as color change [20]. Some studies showed a decrease of procyanidin B2 during conching, but there was an increase of epicatechin and catechin content [34]. Also, Dimick et al. [43] concluded that oxidation of tannins and some enzymatic mechanisms (during tanning process) influence polyphenol content after conching. Complexes between polyphenols, amino acids, peptides, and proteins are formed during this process. This is one of the reasons why conching affects chocolate flavor and reduces astringency [5]. However, Becket et al. [3] state that reactions during conching are not extensive and as important as redistribution of aroma compounds between chocolate particles—in the initial stage (dry phase), aromatic compounds are contained in cocoa particles; however, mixing and shearing redistributes them between sugar, cocoa, and fat, making sugar an aroma-carrier as well.

## 6. Other Components

### 6.1. Methylxanthines

Methylxanthines are alkaloids (theobromine and caffeine) that have stimulatory effects on the central nervous system. Cocoa is the main source of theobromine but has a lower content of caffeine then coffee and tea. The reported values for theobromine and caffeine content in nonfat cocoa bean solids are about 4% and 0.2% of dry weight, respectively [3,33]. Contents can be influenced by the fermentation process and depend on bean type [1,44]. Unfermented cocoa beans often contain theobromine bound to tannins. During fermentation, acetic acid hydrolyzes theobromine–tannin bonds and part of theobromine is released. Free theobromine then diffuses into the cocoa shell [15,45,46].

### 6.2. Aldehydes

Fermentation and drying of cocoa beans produce low concentrations of aldehydes. In further processes of chocolate production, they are important not only for flavor development, but also for further reactions and formation of pyrazines [7,47].

Three aldehydes present in chocolate have strong chocolate aroma: 2-methylpropanal, 2-methylbutanal, and 3-methylbutanal [48]. They are produced during cocoa bean roasting from amino acids, which determine their structure. In chocolate, aldehydes formed by Strecker degradation are also present [5]. Counet et al. [20] reported these were: 2-methylpropanal (derived from valine), 3-methylbutanal (from leucine), 2-methylbutanal (from isoleucine), phenylacetaldehyde (from phenylalanine), and 3-(methylthio)propionaldehyde (from methionine).

**Figure 7:**
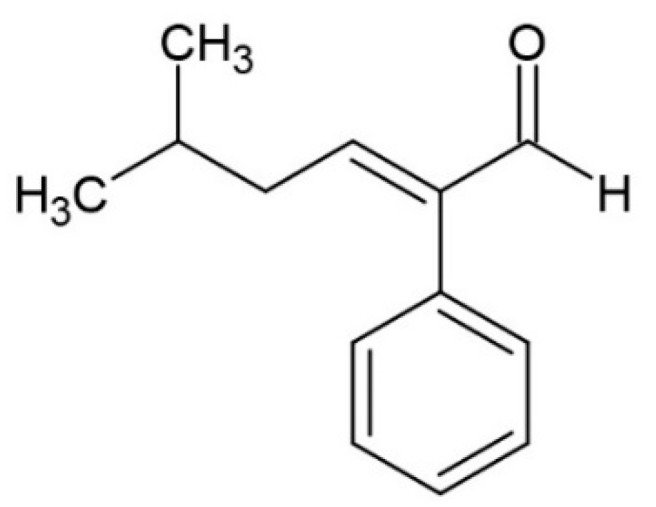
Structure of 2-phenyl-5methyl-2-hexenal.

During conching, some aldehydes are lost due to chemical reactions and evaporation (e.g., concentration of 2-methylpropanal, 2-methylbutanal, and 3-methylbutanal is reduced) and other aldehydes increase through aldol condensation (e.g., 2-phenyl-5-methyl-2-hexenal (Figure 7) formation from phenylacetaldehyde and 3-methylbutanal followed by dehydration) [5,20].

### 6.3. Esters

**Figure 8:**
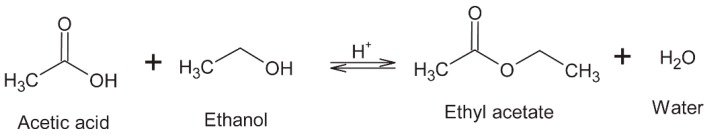
Production of ethyl acetate during fermentation.

Esters are compounds present in unfermented and roasted beans, but can also result from fermentation process (yeast metabolism). One of compounds formed during fermentation is ethyl acetate, which is a product of esterification from ethanol and acetic acid (Figure 8). Other esters present during production are isobutyl acetate, isoamyl acetate, phenylethyl acetate, methyl isopentanoate, and methyl isovalerate [7,49].

The aerobic phase of fermentation is the most important for synthesis of esters from alcohols. Esters are one of the most important volatile compounds because they are associated with fruity flavors [49,50]; however, in chocolate they have secondary role in aroma development, after aldehydes and pyrazines [3].

### 6.4. Ketones

Ketones present in chocolate are 2-heptanone, 2-pentanone, 2-nonanone, acetophenone, and acetoin. These compounds are favorable for cocoa flavor and quality. Ketones are produced during fermentation, and Rodriguez-Campos et al. [47] reported that the longer the fermentation, the higher the content of ketones. Acetoin is the dominant ketone in fermented, dried, roasted beans and cocoa liquor and it is produced by alcoholic fermentation [7,51,52].

### 6.5. Pyrazines

Pyrazines are heterocyclic volatile compounds that give important cocoa flavor and aroma, and were recognized in 1967 when seven alkyl-substituted pyrazines where identified in chocolate aroma: methylpyrazine; 2,3-dimethylpyrazine; 2-ethyl-5-methylpyrazine; trimethylpyrazine; 2,5-dimethyl-3-ethylpyrazine; 2,6-dimethyl-3-ethylpyrazine; and tetramethylpyrazine [53].

**Figure 9:**
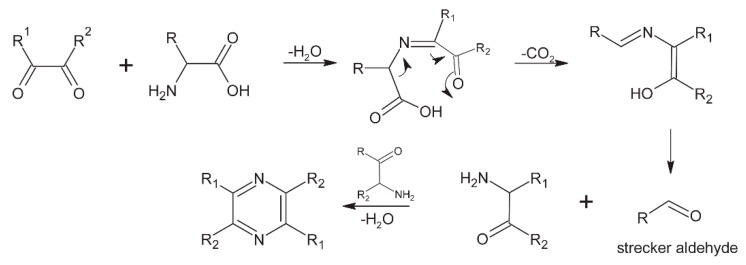
Production of pyrazine.

Tetramethylpyrazine and trimethylpyrazine are the most important for development of nutty and grassy flavor. Most pyrazines are produced during roasting by Maillard reactions and Strecker degradation (Figure 9). The content of these compounds depends on origin of the cocoa beans and the duration and temperature of roasting. It is known that Ghanaian beans have higher content of pyrazines after roasting, mostly because of longer fermentation [3]. The content and ratio of the above-mentioned pyrazines can be good indicators of roasting degree [7,47] because applying increased time and temperature improves production of pyrazines [3]. There are two more ways of pyrazine production: Maillard reactions during drying of beans and as a metabolic product of *Bacillus subtilis* during fermentation [7,54].

### 6.6. Acids

Acids are fermentation products which are produced mainly from sugars. Predominantly, acetic and lactic acids are formed. They reduce the pH to approximately 4.5–5.5, enabling enzymatic reactions necessary in this step of cocoa bean processing. Subsequent fermentation, drying, and roasting significantly reduce the content of volatile acids, which is one of prerequisites for development of the pleasant aroma of the final products [3]. If cocoa beans are dried too quickly, volatile acids are trapped within the bean, which is not desirable, since high acid content leads to off-flavors cocoa and chocolate [7,55]. Low content can be connected to loss of proper flavor of chocolate. Normally, milk chocolate has lower acid content than dark chocolate [56].

### 6.7. Alcohols

Alcohols are present in cocoa and chocolate after fermentation of beans. They are produced mainly by yeasts but some can be formed by combined activity of bacteria and yeast. 2-phenethylethanol and 2,3-butanediol are dominant during all fermentation stages [49,56]. Alcohols are involved in esterification reactions, predominantly with acetic acid, to produce acetates [3].

## 7. Conclusions

The chemistry behind chocolate production is very complex due to large number of involved compounds and processes. The most intensive changes occur during fermentation and roasting of cocoa bean, and during conching of chocolate. These processes must be carefully controlled due to the influence of chemical reactions and their products on chocolate’s flavor and aroma. The main flavor compounds in chocolate are polyphenols, present in raw cocoa bean and going through various forms during production, and pyrazines formed during production, followed by aldehydes, ketones, and esters. Some mechanisms of reactions during cocoa bean and chocolate processing have been revealed and are well-established; however, as new analytical techniques emerge, new compounds are being identified (e.g., seyllo-inositol, 1-kestose and 6-kestose, cacaoic acid) and their involvement in reactions during cocoa bean and chocolate processing and their influence on overall chocolate properties (flavor, aroma, color) are yet to be revealed. Furthermore, as analytical instruments are more and more sensitive in detection and quantification of already identified compounds, new knowledge is expected in this area as well. All this knowledge will contribute not only to science, but to cocoa bean and chocolate producers as well, who will be able to select varieties and target processes to increase contents of desirable compounds and decrease contents of less desirable ones, as well as to optimize ratios of different compounds, which is important for overall quality.

## References

[1] Arunkumar K., Jegadeeswari V. (2019). Evaluating the processed beans of different cocoa (*Theobroma cacao* L.) accessions for quality parameters. J. Phytol..

[2] Gutiérrez T.J. (2017). State-of-the-Art Chocolate Manufacture: A Review. Compr. Rev. Food Sci. Food Saf..

[3] Beckett S.T., Fowler M.S., Ziegler G.R. (2017). Beckett’s Industrial Chocolate Manufacture and Use.

[4] Adeyeye E.I., Akinyeye R.O., Ogunlade I., Olaofe O., Boluwade J.O. (2010). Effect of farm and industrial processing on the amino acid profile of cocoa beans. Food Chem..

[5] Afoakwa E.O., Paterson A., Fowler M., Ryan A. (2008). Flavor Formation and Character in Cocoa and Chocolate: A Critical Review. Crit. Rev. Food Sci. Nutr..

[6] Beckett S.T. (2008). The Science of Chocolate.

[7] Aprotosoaie A.C., Luca S.V., Miron A. (2015). Flavor Chemistry of Cocoa and Cocoa Products-An Overview. Compr. Rev. Food Sci. Food Saf..

[8] Caligiani A., Marseglia A., Palla G. (2016). Cocoa: Production, Chemistry, and Use. Encycl. Food Health.

[9] Bertazzo A., Comai S., Brunato I., Zancato M., Costa C.V.L. (2011). The content of protein and non-protein (free and protein-bound) tryptophan in *Theobroma cacao* beans. Food Chem..

[10] Granvogl M., Bugan S., Schieberle P. (2006). Formation of Amines and Aldehydes from Parent Amino Acids during Thermal Processing of Cocoa and Model Systems: New Insights into Pathways of the Strecker Reaction. J. Agric. Food Chem..

[11] Bytof G., Biehl B., Heinrichs H., Voigt J. (1995). Specificity and stability of the carboxypeptidase activity in ripe, ungerminated seeds of *Theobroma cacao* L.. Food Chem..

[12] Caparosa M.H., Hartel R.W., Melton L., Shahidi F., Varelis P. (2018). Structure and Properties of Chocolate. Encyclopedia of Food Chemistry.

[13] Rottiers H., Tzompa Sosa D.A., De Winne A., Ruales J., De Clippeleer J., De Leersnyder I., De Wever J., Everaert H., Messens K., Dewettinck K. (2019). Dynamics of volatile compounds and flavor precursors during spontaneous fermentation of fine flavor Trinitario cocoa beans. Eur. Food Res. Technol..

[14] Oracz J., Nebesny E. (2014). Influence of roasting conditions on the biogenic amine content in cocoa beans of different *Theobroma cacao* cultivars. Food Res. Int..

[15] Do Carmo Brito B.N., Campos Chisté R., da Silva Pena R., Abreu Gloria M.B., Santos Lopes A. (2017). Bioactive amines and phenolic compounds in cocoa beans are affected by fermentation. Food Chem..

[16] Kongor J.E., Hinneh M., de Walle D.V., Afoakwa E.O., Boeckx P., Dewettinck K. (2016). Factors influencing quality variation in cocoa (*Theobroma cacao*) bean flavour profile—A review. Food Res. Int..

[17] Belitz H.D., Grosch W., Schieberle P. (2009). Food Chemistry.

[18] Oracz J., Nebesny E. (2018). Effect of roasting parameters on physicochemical characteristics of high-molecular-weight Maillard reaction products isolated from cocoa beans of different *Theobroma cacao* L. groups. Eur. Food Res. Technol..

[19] Pontillon J. (1995). La fabrication du chocolat. Pour la Science.

[20] Counet C., Callemien D., Ouwerx C., Collin S. (2002). Use of Gas Chromatography−Olfactometry to Identify Key Odorant Compounds in Dark Chocolate. Comparison of Samples before and after Conching. J. Agric. Food Chem..

[21] Redgwell R.J., Hansen C.E. (2000). Isolation and characterization of cell wall polysaccharides from cocoa (*Theobroma cacao* L.) beans. Planta.

[22] Afoakwa O.E. (2016). Chocolate Science and Technology.

[23] Megías-Pérez R., Ruiz-Matute A.I., Corno M., Kuhnert N. (2018). Analysis of minor low molecular weight carbohydrates in cocoa beans by chromatographic techniques coupled to mass spectrometry. J. Chromatogr. A.

[24] Noor-Soffalina S.S., Jinap S., Nazamid S., Nazimah S.A.H. (2009). Effect of polyphenol and pH on cocoa Maillard-related flavour precursors in a lipidic model system. Int. J. Food Sci. Technol..

[25] Amoako D., Awika J.M. (2016). Polyphenol interaction with food carbohydrates and consequences on availability of dietary glucose. Curr. Opin. Food Sci..

[26] Sirbu D., Grimbs A., Corno M., Ullrich M.S., Kuhnert N. (2018). Variation of triacylglycerol profiles in unfermented and dried fermented cocoa beans of different origins. Food Res. Int..

[27] Sirbu D., Corno M., Ullrich M.S., Kuhnert N. (2018). Characterization of triacylglycerols in unfermented cocoa beans by HPLC-ESI mass spectrometry. Food Chem..

[28] Masuchi Buscato H.M., Hara L.M., Bonomi É.C., Calligaris G.A., Cardoso L.P., Grimaldi R., Kieckbusch T.G. (2018). Delaying fat bloom formation in dark chocolate by adding sorbitan monostearate or cocoa butter stearin. Food Chem..

[29] Wilson P.K., Hurst W.J. (2015). Chocolate and Health—Chemistry, Nutrition and Therapy.

[30] Hurst W.J., Tarka S.M., Dobson G., Reid C. (2001). M Determination of Conjugated Linoleic Acid (CLA) Concentrations on Milk Chocolate. J. Agric. Food Chem..

[31] Cambell W., Drake M.A., Larick D.K. (2003). The Impact of Fortification with Conjugated Linoleic Acid (CLA) on the Quality of Fluid Milk. J. Dairy Sci..

[32] Sonwai S., Rousseau D. (2010). Controlling fat bloom formation in chocolate—Impact of milk fat on microstructure and fat phase crystallisation. Food Chem..

[33] Jalil A., Ismail A. (2008). Polyphenols in Cocoa and Cocoa Products: Is there a Link between Antioxidant Properties and Health?. Molecules.

[34] Zyzelewicz D., Krysiak W., Oracz J., Sosnowska D., Budryn G., Nebesny E. (2016). The influence of the roasting process conditions on the polyphenol content in cocoa beans, nibs and chocolates. Food Res. Int..

[35] De Taeye C., Caullet G., Eyamo Evina V.J., Collin S. (2017). Procyanidin A2 and Its Degradation Products in Raw, Fermented, and Roasted Cocoa. J. Agric. Food Chem..

[36] Bonvehí J.S. (2005). Investigation of aromatic compounds in roasted cocoa powder. Eur. Food Res. Technol..

[37] Emmanuel O.A., Jennifer Q., Agnes S.B., Jemmy K.S.T., Firibu K.S. (2012). Influence of pulp-preconditioning and fermentation on fermentative quality and appearance of Ghanaian cocoa (*Theobroma cacao*) beans. Int. Food Res. J..

[38] Kothe L., Zimmermann B.F., Galensa R. (2013). Temperature influences epimerization and composition of flavanol monomers, dimers and trimers during cocoa bean roasting. Food Chem..

[39] Quiroz-Reyes C.N., Fogliano V. (2018). Design cocoa processing towards healthy cocoa products: The role of phenolics and melanoidins. J. Funct. Foods.

[40] Ioannone F., Di Mattia C.D., De Gregorio M., Sergi M., Serafini M., Sacchetti G. (2015). Flavanols, proanthocyanidins and antioxidant activity changes during cocoa (*Theobroma cacao* L.) roasting as affected by temperature and time of processing. Food Chem..

[41] Stanley T.H., Van Buiten C.B., Baker S.A., Elias R.J., Anantheswaran R.C., Lambert J.D. (2018). Impact of roasting on the flavan-3-ol composition, sensory-related chemistry, and in vitro pancreatic lipase inhibitory activity of cocoa beans. Food Chem..

[42] Hurst W.J., Krake S.H., Bergmeier S.C., Payne M.J., Miller K.B., Stuart D.A. (2011). Impact of fermentation, drying, roasting and Dutch processing on flavan-3-ol stereochemistry in cacao beans and cocoa ingredients. Chem. Cent. J..

[43] Dimick P.S., Hoskin J.C., Beckett S.T. (1999). The Chemistry of Flavour Development in Chocolate. Industrial Chocolate Manufacture and Use.

[44] Todorovic V., Redovnikovic I.R., Todorovic Z., Jankovic G., Dodevska M., Sobajic S. (2015). Polyphenols, methylxanthines, and antioxidant capacity of chocolates produced in Serbia. J. Food Compost. Anal..

[45] Barišić V., Flanjak I., Križić I., Jozinović A., Šubarić D., Babić J., Miličević B., Ačkar Đ. (2019). Impact of high-voltage electric discharge treatment on cocoa shell phenolic components and methylxanthines. J. Food Process. Eng..

[46] Williams O.B., Mark E.M., Stewart G.F. (1958). Advances in Food Research.

[47] Rodriguez-Campos J., Escalona-Buend’ıa H.B., Contreras-Ramos S.M., Orozco-Avila I., Jaramillo-Flores E., Lugo-Cervantes E. (2012). Effect of fermentation time and drying temperature on volatile compounds in cocoa. Food Chem..

[48] Voigt J., Janek K., Textoris-Taube K., Niewienda A., Wöstemeyer J. (2016). Partial purification and characterisation of the peptide precursors of the cocoa-specific aroma components. Food Chem..

[49] Hamdouche Y., Meile J.C., Lebrun M., Guehi T., Boulanger R., Teyssier C., Montet D. (2019). Impact of turning, pod storage and fermentation time on microbial ecology and volatile composition of cocoa beans. Food Res. Int..

[50] Moreira I.M.V., Vilela L.F., Santos C., Lima N., Schwan R.F. (2018). Volatile compounds and protein profiles analyses of fermented cocoa beans and chocolates from different hybrids cultivated in Brazil. Food Res. Int..

[51] Pretorius I.S. (2000). Tailoring wine yeast for the new millennium: Novel approaches to the ancient art of winemaking. Yeast.

[52] Menezes A.G.T., Batista N.N., Ramos C., Silva A.R.A., Efraim P., Pinheiro A.C.M., Schwan R.F. (2016). Investigation of chocolate produced from four different Brazilian varieties of cocoa (*Theobroma cacao* L.) inoculated with *Saccharomyces cerevisiae*. Food Res. Int..

[53] Damodaran S., Parkin K.L. (2017). Fennema’s Food Chemistry.

[54] Ramli N., Hassan O., Said M., Samsudin W., Idris N.A. (2006). Influence of roasting conditions on volatile flavor of roasted Malaysian cocoa beans. J. Food Process Press.

[55] Ascrizzi R., Flamini G., Tessieri C., Pistelli L. (2017). From the raw seed to chocolate: Volatile profile of *Blanco de Criollo* in different phases of the processing chain. Microchem. J..

[56] Liu J., Liu M., He C., Song H., Guo J., Wang Y., Yang H., Su X. (2014). A comparative study of aroma-active compounds between dark and milk chocolate: Relationship to sensory perception. J. Sci. Food Agric..

